# The Hematopoietic Niche in Myeloproliferative Neoplasms

**DOI:** 10.1155/2015/347270

**Published:** 2015-11-30

**Authors:** Annette H. Schmitt-Graeff, Roland Nitschke, Robert Zeiser

**Affiliations:** ^1^Department of Pathology, Freiburg University Medical Center, Albert-Ludwigs University, 79106 Freiburg, Germany; ^2^Life Imaging Center, Center for Biological Systems Analysis (ZBSA), Albert-Ludwigs University, 79104 Freiburg, Germany; ^3^Department of Hematology and Oncology, Freiburg University Medical Center, Albert-Ludwigs University, 79106 Freiburg, Germany

## Abstract

Specialized microanatomical areas of the bone marrow provide the signals that are mandatory for the maintenance and regulation of hematopoietic stem cells (HSCs) and progenitor cells. A complex microenvironment adjacent to the marrow vasculature (vascular niche) and close to the endosteum (endosteal niche) harbors multiple cell types including mesenchymal stromal cells and their derivatives such as CAR cells expressing high levels of chemokines C-X-C motif ligand 12 and early osteoblastic lineage cells, endothelial cells, and megakaryocytes. The characterization of the cellular and molecular networks operating in the HSC niche has opened new perspectives for the understanding of the bidirectional cross-talk between HSCs and stromal cell populations in normal and malignant conditions. A structural and functional remodeling of the niche may contribute to the development of myeloproliferative neoplasms (MPN). Malignant HSCs may alter the function and survival of MSCs that do not belong to the neoplastic clone. For example, a regression of nestin^+^ MSCs by apoptosis has been attributed to neuroglial damage in MPN. Nonneoplastic MSCs in turn can promote aggressiveness and drug resistance of malignant cells. In the future, strategies to counteract the pathological interaction between the niche and neoplastic HSCs may offer additional treatment strategies for MPN patients.

## 1. Introduction

The quiescence, self-renewal, and fate determination of hematopoietic stem cells (HSC) and progenitor cells (HPC) depend on their close interaction with the supportive bone marrow (BM) microenvironment and the tight interaction with multiple cellular and matrix components, adhesion molecules, chemokines, and their receptors, as well as soluble and membrane-bound factors present in the BM stroma of adults [1, 2]. Moreover, there is emerging evidence that the nervous system and oxygen tension in the microenvironment have an impact on hematopoiesis [2]. The network of these constituents is not equally distributed throughout the BM spaces but is mainly localized in specialized areas referred to as “niches.” The term “hematopoietic (HSC) niche” was proposed by Schofield in 1978 to designate “an entity in which the stem cell's maturation is prevented and the properties of ‘stemness' are preserved” [3].

The adult HSC niche is traditionally subdivided in different microenvironmental compartments that harbor BM mesenchymal stromal cells (MSCs) and their progeny in close association with HSCs/HPCs (HSPCs). The vascular/perivascular niche is localized in the area of small caliber arterioles and sinusoids where HSPCs are controlled by the so-called close proximity signals from endothelial cells, MSCs, and megakaryocytes [2, 4–8]. The components of the vascular niche control HSC maintenance, cell cycle, and trafficking activity. The osteoblastic or endosteal niche resides close to the endosteal surface of the bone trabeculae that are lined by osteoblasts/early osteoblastic lineage cells (OBCs) which are considered to be a key component of the endosteum. OBCs have been mainly implicated in the regulation of B-lymphopoiesis and may contribute to in HSC quiescence [2, 3, 7]. In vivo findings and experimental studies suggest that quiescent HSCs in the G0/G1 phase mainly reside in periphery of the bone marrow spaces close to the trabecular bone while HPCs committed to proliferation and differentiation localize to the central parts of the BM [9]. However, the close proximity between components of the BM vasculature such as small arterioles and sinusoids to the endosteal bone surface suggests functional interactions of both specialized microanatomical areas. Furthermore, observations obtained from murine models and human specimens point to a conceptual framework including elements of the vascular and the osteoblastic niches. According to the definition given by Schepers et al., the cellular components of the niche can be divided into two functional types: (a) essential cell types that provide close proximity signals to HSCs and (b) accessory cells that have long-range and often indirect influences on HSCs [2]. However, the discrimination between these two types remains a matter of controversial discussion, especially since their function may not be stable but may be modulated in different physiologic and pathologic conditions.

Unravelling the complex relationships between HSPCs and the specialized microenvironment will continue to provide important insights in the regulation of normal and neoplastic hematopoiesis. Pathways of the cross-talk between malignant cells and their microenvironment may offer treatment approaches in myeloid malignancies similar to advanced targeted therapeutics in chronic lymphocytic leukemia [10]. Many data obtained from experimental studies are beyond the scope of this review. Our aim is to provide a short overview on the HSC niche and its emerging role in myeloproliferative neoplasms (MPN) that we illustrate by some of our observations concerning the in situ localization of stromal components in BM trephine biopsies of MPN patients.

## 2. The Composition of HSCs Niches in the Normal Bone Marrow

### 2.1. Mesenchymal Stromal Cells

**Figure 1:**
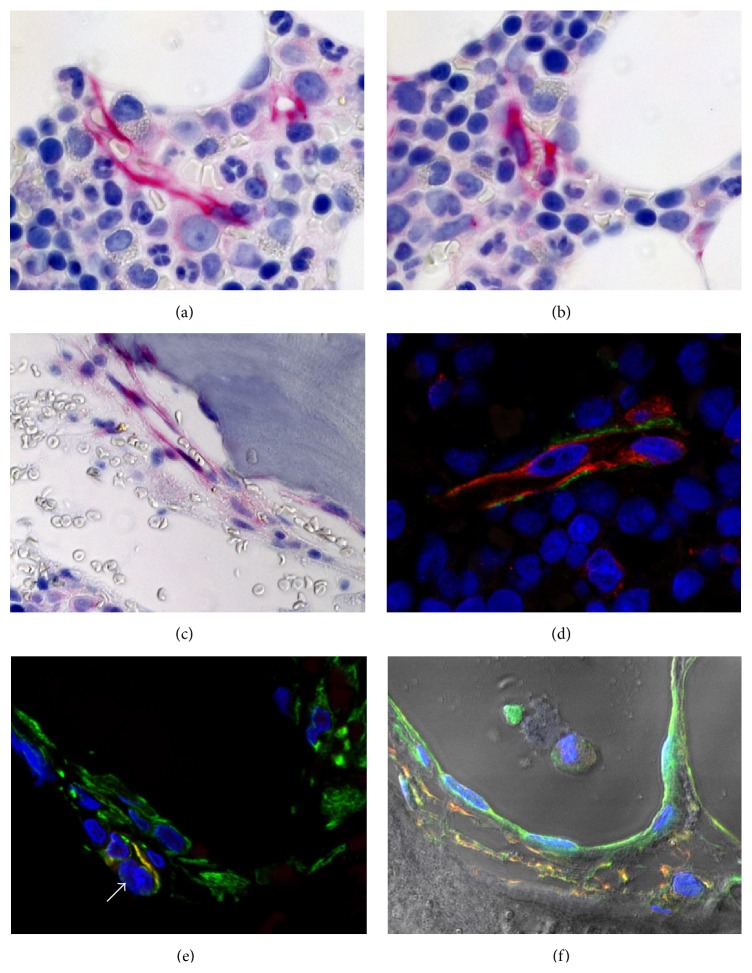
Trephine BM biopsy obtained from a healthy control studied by immunohistochemistry (red) and immunofluorescence techniques (red, green, and coexpression signals: yellow) and visualized using brightfield (a–c) or laser scanning microscopy (LSM, (d–f)). (a, b, c) Nes^+^ cells (red) near arteriolar and sinusoidal blood vessels of the central BM areas (a, b) and (c) close the trabecular bone. (d) Localization of Nes^+^ (green) and CD146^+^ (red) cells around a small sinusoidal vessel in the central part of the BM. (e, f) Strong expression of CXCL12 (green) by endothelial and perisinusoidal cells that partially coexpress ((d), yellow) nestin (red) and synemin (green). Note perivascular CAR cell in (e) (arrow).

Despite numerous studies, the characterization of human bone marrow MSCs and of the hematopoietic niche in vivo especially in the human BM (Figure 1) remains challenging [11]. The knowledge of the immunophenotype of human MSCs is still limited [2, 8]. As recently discussed, the term MSC is often utilized for heterogeneous stromal populations containing few primitive stem cells and abundant cells with properties of primary fibroblasts [8]. MSCs are BM-derived nonhematopoietic precursor cells that have the capacity to renew themselves, to differentiate into other mesenchymal cells including OBCs, fibroblast-like stromal cells, and fat cells, and to engraft in injured organs [2, 11, 12]. Comparative gene expression analysis has identified a low/negative PDGFR*α* (CD140a) expression in a close to pure population of BM marrow stromal/progenitor cells [13]. MSCs that contain high levels of the chemokines C-X-C motif ligand 12 (CXCL12) have been designated as CAR cells [2, 3, 14]. CAR cells are predominantly localized in the perivascular area near sinusoids and arterioles but may also be observed in the vicinity of trabecular bone. The transcription factor Foxc1 is preferentially expressed in the adipoosteogenic progenitor CAR cells essential for HSPC maintenance in vivo [15].

The perivascular MSC are closely related to pericytes as suggested by the detection of the pericyte marker neuron/glial antigen 2 (NG2) [2]. Perivascular MSC subpopulations expressing variable amounts of PDGFRa or CD140a, CD51, Sca-1, leptin receptor (LEP-R), nestin (Nes), and CD146 are considered to be important cellular players of the stem cell niche [2, 5, 6, 14, 16]. It has been reported that 60% of HSCs are directly associated with the vasculature [2].

### 2.2. Vascular Endothelial Cells and Megakaryocytes

Other well characterized critical cellular components of the vascular niche are CD31^+^CD34^+^ endothelial cells and CD41^+^CD61^+^ megakaryocytes [2, 17]. The vascular endothelium especially of the small arterioles and sinusoids communicates with the HSCs through direct contact and multiple soluble and membrane-bound factors including E-selectin [2, 3]. Endothelial cells that express among many other molecules CXCL12 are considered to contribute to HSC regulation and retention in the niche [2]. Megakaryocytes are the source of CXCL4 and conferring quiescence of HSCs and regulating the HSC pool size [17]. Induction of HSCs quiescence by megakaryocytes has also been attributed to thrombopoietin [18].

### 2.3. Osteoblastic Lineage Cells

OBCs are localized in the osteoblastic niche near the endosteum. The contribution of OBCs to HSC maintenance is still controversially discussed [19]. While some authors consider OBCs as accessory components of the HSCs niche [2], work from other groups suggests that osteoblasts directly regulate HSCs and their migration [20, 21]. The gap junction protein connexin-43 (Cx43) is highly expressed in OBCs and is critical for the composition of the microenvironment and the lodging and mobilization of HSC/Ps in nonmyeloablated animals [22]. The ablation of developing OBCs has been shown to result in a loss of lymphoid, erythroid, and myeloid progenitors [22]. In an ex vivo culture system, OBCs are able to support hematopoiesis [21]. Other studies have suggested that osteoblasts may only create a niche for certain early lymphoid progenitors but not for HSCs [7]. The transcription factor Runx2 is involved in the differentiation of osteoblasts and the induction of osteopontin [23]. The activation of PTH/PTHrP receptors leads to an increase in osteoblasts and an activation of the Notch ligand, jagged 1 on osteoblasts, and favors an expansion of HSCs [24, 25]. Osteoblasts express several members of the Wnt signaling molecules [1]. In experimental studies, Wnt signaling as well as bone morphogenetic protein and early B-cell transcription factor control the hematopoietic niche [1, 26]. Apparently, an interaction of Wnt and Notch signaling pathways plays an important role. Wnt signaling is implicated in the balance between HSC-renewal and differentiation and in the disruption of this balance in hematopoietic neoplasms [1, 27]. Notch is essential for the generation of HSCs and an essential regulator of hematopoietic differentiation [28].

### 2.4. Sympathetic Neurons and Schwann Cells

Schepers et al. have listed sympathetic nerve fibers surrounded by nonmyelinating Schwann cells among the accessory components of the niche [2]. A so-called long-range indirect influence on the HSC niche has been attributed to these populations [2, 29]. However, several publications show that sympathetic neurons together with the associated Schwann cells apparently do not play a mere accessory role in the niche [19, 30–33]. In the developing BM HSC niche forming nestin^+^ MSCs share a common origin with sympathetic peripheral neurons and glial cells [31]. All three cell types have been referred to as neural crest derived regulators of adult HSCs activity [31]. Autonomic nerves that are sheeted by glial cells and express HSCs niche factor genes were shown to be in contact with a substantial proportion of HSCs and to regulate the activation process of TGF-*β* [19]. Autonomic nerve denervation reduced the number of these active TGF-*β*-producing cells and led to rapid loss of HSCs from BM [19]. Sympathetic nerve fibers are also involved in the circadian regulation of noradrenalin secretion and CXCL12 expression by perivascular MSCs and in the mobilization of HSCs [5, 30]. Moreover, signals from the nervous system are involved in regulation of steady state egress and rapid mobilization of hematopoietic progenitor cells [32]. In MPN, HSCs carrying a* JAK2* V617F mutation induce neuroglial damage and Schwann cell death that contributes the pathogenesis of the disease [33]. It has even been proposed that targeting the neural regulation of the HSCs niche may be a therapeutic option in MPN [33].

### 2.5. Monocyte/Macrophages

Monocytes and macrophages, especially bone-associated macrophages, contribute to the regulation of the activity BM niche cells and the maintenance of HSPCs [2, 19, 34]. Macrophages modulate CXCL12 expression by nestin-GFP^+^ cells and HSC retention in the bone marrow following a circadian rhythm [20]. Monocyte lineage cells in the bone marrow contribute to osteoblast homeostasis and HSPC trafficking [35]. G-CSFR signaling in monocytic cells is sufficient to induce HSPC mobilization [35]. However, the role of multinucleated osteoclasts is controversially discussed. In mice, this population is not required for the efficient retention of HSPCs in the bone marrow and is dispensable for HSPC mobilization by G-CSF [36].

### 2.6. The Endosteal Niche

Calcium ions, reactive oxygen species, and oxygen tension contribute to the function of the endosteal niche. Transplanted hematopoietic stem/progenitor cells preferentially localize to blood vessels in the endosteal area, a phenomenon that may result from high local concentrations of ionic calcium [20].

The endosteal niche is a hypoxic area of the marrow cavities where accumulation of the transcription factor HIF-1*α* probably may contribute to the homing of HSPCs. Important cellular components of the endosteal niche include endothelial cells, macrophages, and MSCs around abundant blood vessels. The MSC subpopulation present near the trabecular bone functionally and phenotypically differs from those from other MSCs subsets [20, 37]. This hypoxic endosteal region harbors MSCs showing a weak or absent CD146 expression probably due to low oxygen and high calcium levels [37]. CD146^+^ osteoprogenitor cells are able to direct ectopic bone formation accompanied by hematopoietic seeding [38]. CAR cells coexpressing high levels of CD146 are mainly found in perivascular regions [37]. However, CXCL12^high^ CAR cells and Nes^+^ MSC-like cells are not restricted to the perivascular niche but were found in the OBC fraction together with osterix (Osx)^+^ osteoprogenitors in GFP reporter mice [39]. Most Osx-GFP^+^ cells were demonstrated at the bone surface while CXCL12-GFP^high^ and NesGFP^+^ cells were present at the bone surface and throughout the BM cavity [39, 40].

### 2.7. The Vascular/Perivascular Niche

The vascular/perivascular niche is localized in the area of sinusoids and small caliber arterioles [23, 41]. This area is seeded by perivascular MSCs expressing the chemokine CXCL12 that is an important regulator of HSC mobilization and a HSCs chemoattractant through its receptor CXCR4 [3, 14, 20, 40–42]. The perivascular populations include CXCL12-abundant CAR cells and Nes^+^ and Lepr^+^ stromal cells. CAR cells are involved in HSCs and B-lymphoid progenitor maintenance [14, 16]. Considerable advances concerning the concept of the HSC niche are derived from experimental studies using Nes-Gfp transgenic mice [5]. Nes^+^ MSCs have been shown to be closely associated with HSCs and are required for HSPC homing after transplantation into lethally irradiated mice [5]. CD45^−^ Nes-GFP^+^ cells have the capacity to form the fibroblastic colony forming unit (CFU-F) and multipotent mesenchymal spheres (mesenspheres) [5, 8].

Human mesenspheres express nestin and Lepr [8]. This and other experimental studies provided evidence that Nes^+^ cells that are innervated by sympathetic neurons are an important component of the HSCs niche [8, 33, 42]. It has recently been proposed that hematopoietic and neurogenic niches share regulatory pathways putatively resulting from the presence of neural crest derivatives in the BM [43]. MSCs containing the intermediate filament type nestin have been considered as neural-crest derived cells (NCSC) [31, 33, 43]. The high-molecular-weight cytoskeletal protein nestin belongs together with synemin to type VI of intermediate filaments [44]. Both nestin and synemin are predominantly expressed in stem and progenitor cells but also in endothelial cells and muscle cells, respectively [44].

Imaging analyses of HSCs niches have shown that quiescent HSCs are associated with small caliber arterioles. Therefore, the existence of 2 functionally distinct vascular niches has been proposed: an arteriolar niche promoting quiescence NG2^+^Nes^bright^ periarteriolar cells and a Lepr^+^Nes^dim^ sinusoidal niche containing mobilizable and proliferative HSCs/HPCs [25]. The sinusoidal niche plays a critical role in hematopoietic cell migration and homing via adhesion molecules such as vascular adhesion molecule-1 (VCAM-1) and selectins [3, 23, 45].

The strict separation between an endosteal and a vascular/perivascular niche is still discussed, since vascular structures are present close to the bone trabeculae and may influence the HSCs in both niches [37, 38].

Experimental high resolution live imaging studies have recently pointed to dynamic niche interactions upon HSCs colonization rather than to a static number of niches [46]. Examples include the rapid remodeling of endothelial cells around a stem cell thus retaining and protecting the new arrivals [47].

Our in situ observations in human BM biopsies show that nestin^+^ CXCL12^+^ cells suggestive of CAR cells and CD146^+^ stromal cells are present near the vascular/perivascular niches in the central parts of the BM cavity and near the trabecular bone (Figure 1).

### 2.8. The HSC Niche in Myeloproliferative Neoplasms

There is increasing evidence that the hematopoietic niche influences leukemic stem cell proliferation, survival, and migration [3, 8, 30, 39, 46, 48]. The BM niche undergoes a profound modulation secondary to the neoplastic transformation of HSC/HPCs in MPN. The disruption of the physiologic architecture of the bone marrow in myeloid neoplasms indicates profound alterations of the bone marrow niches [39]. Examples include the description of an activated fibronectin-secretory pathway in stromal cells of prefibrotic MPN or JAK-STAT pathway activation in malignant and nonmalignant cells in MPN [30, 49, 50]. A modulation of the bone marrow niche may even contribute to the transformation of normal HSCs into leukemic stem cells (LCS) [1].

Alteration of the BM microenvironment by osteoblastic cell-specific activation of the parathyroid-hormone receptor suppresses BCR-ABL1-mediated MPN and may impair the maintenance of LSCs in mouse transplantation models [45].

Experimental studies performed in a double-transgenic mouse model of chronic phase CML have suggested that leukemic myeloid cells remodel the endosteal BM niche [31]. The leukemic niche contains functionally altered OBCs, impairs normal hematopoiesis, favors LSC function, and contributes to the development of myelofibrosis [39]. In this model of chronic phase CML, a large increase in OBCs has been reported to be associated with an accumulation of myelofibrotic cells and increased collagen deposition and expansion of trabecular bone [39]. The overproduction of OBCs may result from direct interactions between MSCs and neoplastic cells as well as by thrombopoietin and the chemokine (C-C motif) ligand 3 (CXCL3) [39]. Schepers et al. also demonstrated that changes in transforming growth factor-*β* (TGF-*β*), Notch, and inflammatory signaling contribute to the environment that promotes the modulation of MPN-expanded OBCs into inflammatory myelofibrotic cells [39]. Additional results obtained in this model indicate that the ability of MPN-expanded OBCs to maintain normal but not LSC is severely compromised. This phenomenon apparently results from a downregulation of the quiescence-enforcing* Tgfb1* and an upregulation of the myeloid-promoting* Tgfb2* [39]. Moreover, a decreased CXCL12 expression by OBCs may enhance the mobilization and loss of normal HSCs in MPN [14, 45].

Krause et al. have shown that BCR-ABL1-expressing LSCs are more dependent on selectins and their ligands as well as on the type I transmembrane glycoprotein and adhesion molecule CD44 for homing and engraftment than normal HSCs [45]. The interactions of CD44 with the extracellular matrix components especially hyaluronic acid may be of special importance for the retention of LSCs in the niche [51–53]. The niche provides a sanctuary for LSCs subsets where they may evade chemotherapy and acquire resistance [52]. Moreover, infiltration of the preexisting niche areas and disruption of the regular structures by LSCs may contribute to the impairment of normal hematopoiesis in myeloid malignancies. A therapeutic approach in an AML-mouse model using an anti-CD44 antibody markedly reduced leukemic repopulation indicating that a disruption of the interaction between LSCs and niche components may offer therapeutic strategies to eliminate quiescent AML LSCs [53]. It has recently been postulated that the leukemia-induced remodeling of the BM microenvironment including altered expression of CXCL12 and JAG1 may be an intrinsic part of leukemogenesis and support chemoresistance [54]. Strategies to target the LSC in its niche are actually discussed [33, 45, 52–55]. Another aspect of the disturbed interaction between LSCs and the BM niche is the observation that LSCs may be less dependent on survival and proliferation signals of the microenvironment than their nonneoplastic counterpart due to autonomous growth signals [2, 56]. These findings may explain the presence of LSCs outside the traditional niche areas both in the BM and in extramedullary sites. Here, LSCs or progenitors that acquire the ability of self-renewal and proliferation may form mass lesions designated as myeloid sarcomas in patients with myelodysplastic syndrome, AML, and MPN or even without overt BM involvement by a malignant population.

In the 2008 World Health Organization (WHO) classification of haematopoietic and lymphoid neoplasms, eight different entities are listed in the MPN category [57]. The diagnostic criteria for these disorders will be updated in the 2016 edition. The classical Philadelphia-chromosome negative (Ph^−^) MPNs comprise the multipotent stem cell disorders polycythemia vera (PV), essential thrombocythemia (ET), and primary myelofibrosis (PMF) that are characterized by a characteristic spectrum of mutations [57–59]. The BM and extramedullary sites such as the spleen show an expansion of precursors of one or several hematopoietic lineages. The proliferation of the myeloid, erythroid, and megakaryocytic cell lineages are generally accompanied by an increase in the respective blood components, red and/or white blood cells and/or platelets. Approximately 95% of PV cases harbor JAK2* V617F* or rarely* JAK2* exon 12 mutations. The frequency of a somatic JAK2* V617F* mutation ranges within about 50% to 60% in ET and PMF while mutations in calreticulin occur within about 20% to 35% of patients [59]. Mutations in the thrombopoietin receptor* MPL* are reported in 3% to 8% of ET and PMF [59]. ET and PMF cases with none of these three “driver” mutations have been designated as “triple negative” [59].

A common feature observed in PMF, a subset of PV and in a minority of ET, is a tendency towards progressive remodeling of the bone marrow stroma resulting in fibrosis and osteosclerosis as a hallmark of a profound alteration of the microenvironment including the niche. Grading of bone marrow fibrosis in MPN by hematopathologists is widely used and will currently be refined [60]. Clinicopathological studies have shown that BM fibrosis regresses in a high percentage of MPN patients after reduced-intensity conditioning allogeneic stem cell transplantation (allo-SCT) and results in a favorable survival independent of IPSS risk score at transplantation [61].

**Figure 2:**
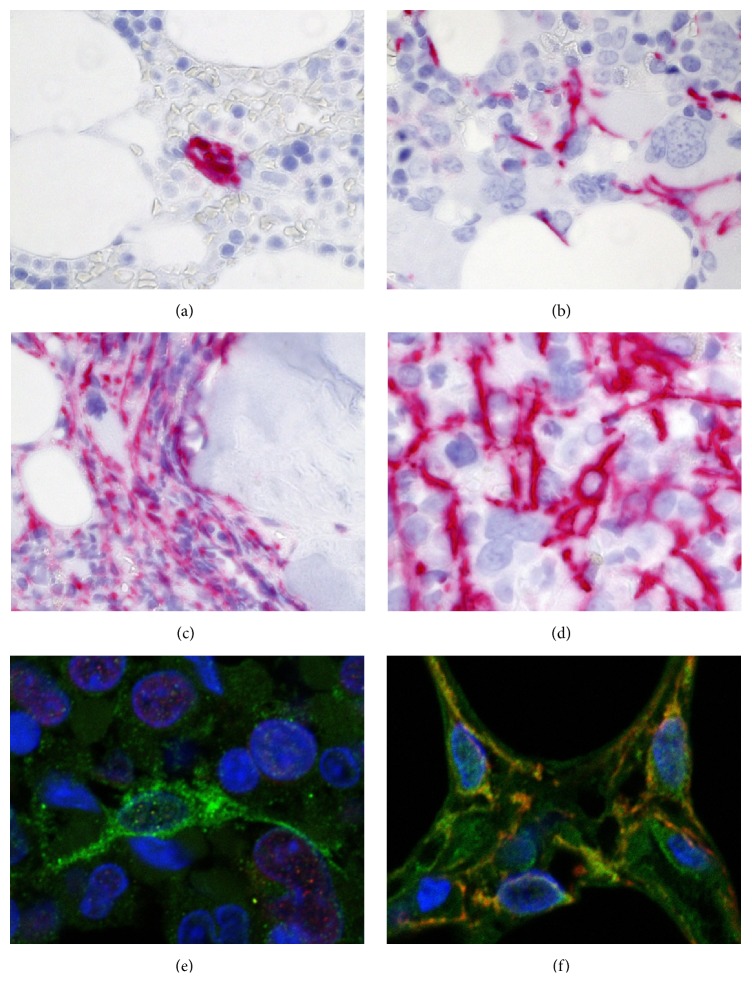
Patterns of *α*-SMA expression in human BM biopsies. (a) *α*-SMA^+^ pericytes (red) surrounding a small arteriolar vessel in a healthy control. (b) Scattered *α*-SMA^+^ stromal cells (red) within a cluster of atypical megakaryocytes in PMF-1. (c, d) BM of a patient with PMF-3: striking expansion of *α*-SMA^+^ cells (red) in (c) the endosteal fibrotic areas of the bone marrow cavity and in (d) the vicinity of clusters formed by megakaryocytes. (e, f) LSM imaging of BM biopsies labelled by anti-*α*-SMA (green) and anti-CRBP-1 (red) antibody (e) shows no colocalization of both antigens in ET myofibroblasts but (f) highlights coexpression (yellow) in abundant myofibroblasts in PMF-3.

Structural changes of the BM architecture reflect a disturbed interaction between the neoplastic HSCs and the nonneoplastic microenvironment. To highlight the presence of cellular components of the hematopoietic microenvironment including the niche, we performed imaging studies by conventional light and laser scanning microscopy on paraffin-embedded EDTA-decalcified trephine BM biopsies obtained from patients with ET, PMF, and PV according to the ethical standards of our institutions. Multiple cellular and soluble factors favor the recruitment myofibroblasts that overexpress *α*-smooth muscle actin (*α*-SMA) [62]. In fibrotic MPN, persistent myofibroblasts that are abundant in the endosteal and perivascular areas contribute to BM fibrosis by excessively producing collagenous extracellular matrix and may thus profoundly alter the HSC niche (Figure 2).

**Figure 3:**
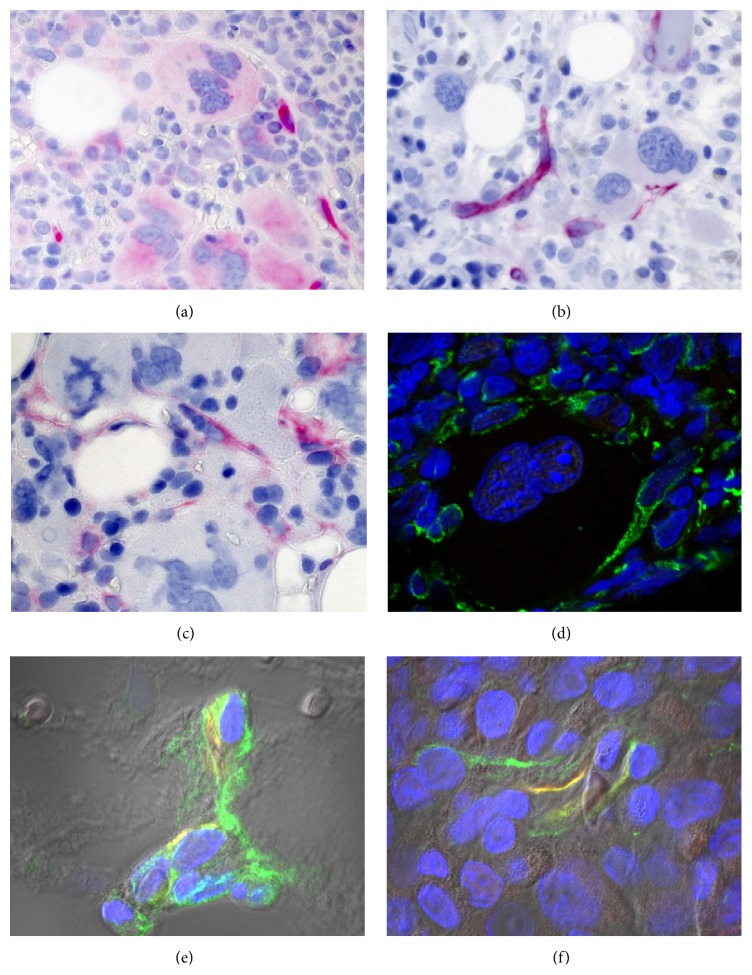
BM stromal cells suggestive of niche components in patients with (a) ET or (b–f) PMF with fibrosis grade 1 (PMF-1). (a, b) Nes^+^ (bright red) as well as (c) CD146^+^ (red) and (d) CXCL12^+^ (green) stromal cells in the vicinity of abundant multinucleated megakaryocytes. (e, f) Coexpression (yellow) of CXCL12 (green) and nestin (red) by perivascular (e) or interstitial stromal cells.

**Figure 4:**
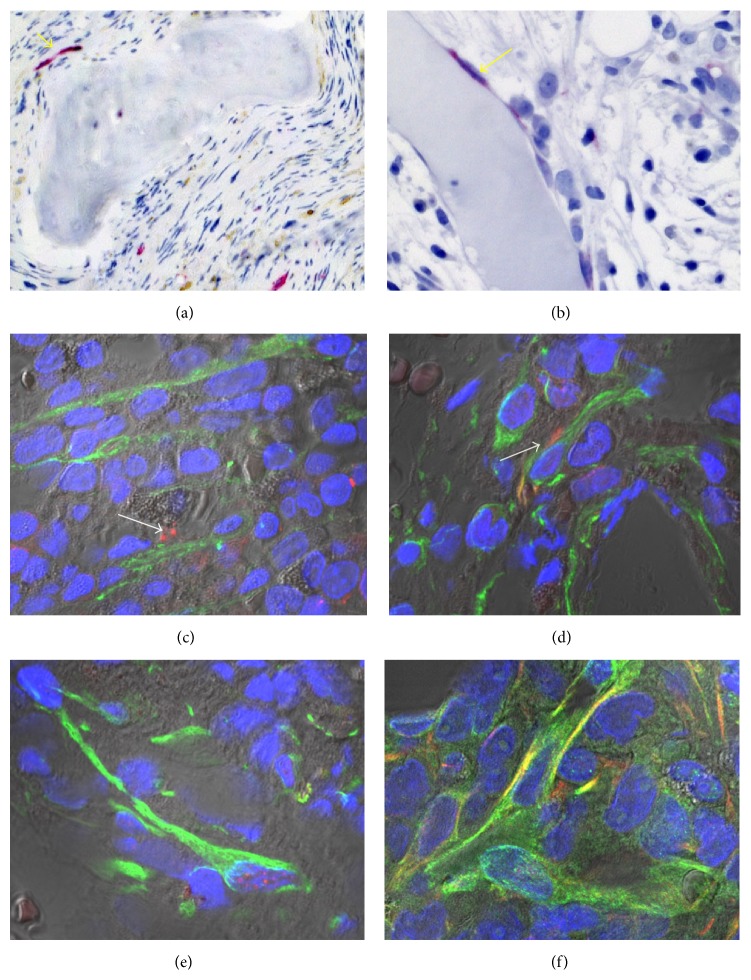
The BM microenvironment as visualized in PMF with high grade reticulin and collagen fibrosis (PMF-3) accompanied by osteosclerosis. Absence of Nes^+^stromal cells in the dense fibrous tissue rimming the bone trabeculae while some Nes^+^ endothelial cells (red, arrows) of (a) arterioles or (b) sinusoids are still present. (c–f) Numerous CXCL12^+^ (green) stromal cells partially coexpressing (f) synemin (red, coexpression in yellow). (c, e) scattered Nes^+^ cytoplasmic fragments (arrows) suggestive of apoptotic cellular remnants.

The cross-talk is reciprocal: the neoplastic cell population influences the stromal cells by the production of various growth factors and cytokines [63, 64]. Megakaryocytes and monocytes/macrophages are considered to be critical in the context of promoting BM fibrosis. Moreover, the microenvironment containing newly formed vessels, inflammatory cells, and profibrotic cytokines such as TGF-*β*1 may contribute to conversion of MSCs in contractile *α*-SMA^+^ myofibroblasts that are abundant in MPN developing overt BM fibrosis [65]. This phenomenon is also observed in cancer-associated myofibroblasts that at least partially develop from BM-derived MSCs [66]. A recently published experimental study performed on bone-marrow derived human MSCs has shown that *α*-SMA-positive human MSCs exhibit low self-renewal and lineage differentiation potential, in contrast to *α*-SMA-negative MSCs, which are clonal and multipotent [66]. Increased expression of *α*-SMA directly guided MSC differentiation towards osteoblasts [66]. These findings suggest that the development of *α*-SMA^+^ myofibroblasts in fibrotic MPN may also disturb the differentiation of MSCs into the characteristic cellular niche components. As discussed above, actual therapeutic concepts including allo-SCT can result in a regression of fibrosis that is associated with a reduction of SMA^+^ myofibroblasts (data not shown). Interestingly, cultured human *α*-SMA^+^ MSC can be deactivated to loose fibrotic MF features and regain their lineage potential [66]. SMA^+^ myofibroblasts in the advanced fibrotic may also coexpress cellular retinol-binding protein 1 (CRBP-1) that is a key component of the retinoid signaling pathway. An experimental study performed on rat BM cells has provided evidence that CRBP-1 overexpression promoted osteogenic differentiation of MSCs through inhibiting RXR*α*-induced *β*-catenin degradation [67]. Nes^+^ MSCs that are innervated by sympathetic nerve fibers in bone marrow niches are considered as key players in the regulation of HSCs. This MSC subset is an important topic area of research in MPN [5, 8, 33]. Arranz et al. have shown that Nes^+^ MSCs, sympathetic nerve fibers, and Schwann cells are reduced in the bone marrow of MPN patients and in mice whose HSCs carry the JAK*V617F* mutation [33]. Apparently, the reduction of Nes^+^ cells results from apoptotic cell loss secondary to sympathetic neuropathy that sensitizes Nes^+^ cells to the cytokine interleukin-1*β* produced by the neoplastic HPCs [33]. The depletion of Nes^+^ cells or their production of CXCL12 contributed to an expansion of JAK2*V617F*-mutated HSCs and a progression of MPN [33]. Figures 3 and 4 demonstrate examples of human BM biopsies immunolabeled by a range of antibodies to highlight different stromal cell populations.

The BM microenvironmental abnormalities may not only contribute to the pathogenesis of myeloid neoplasms but also have a negative impact on treatment response.

Observations obtained from an in vitro culture system have suggested that stroma-derived cytokines may protect JAK2*V617F*-mutated cells against JAK2-inhibitor therapy [68]. However, clinical therapeutic strategies using long-term JAK2-inhibitors resulted in a marked modulation of the BM cellular microenvironment. The effect was seen across all aspects of the fibrotic process including the inflammatory BM stromal reaction associated with MF [69]. These observations are an important step to a better understanding of the interactions between the microenvironment of the HSC niche and malignant myeloid cells in MPN.

## 3. Conclusion and Perspectives

The hematopoietic niches may be considered as an “ecosystem” that provides signals for the maintenance and regulation of HSPCs. In hematologic malignancies, the stromal compartment of the BM does not belong to the neoplastic clone.

However, there is evidence of an ongoing cross-talk between the nonneoplastic BMSCs present in the niches and the malignant cells that may result in a modulation of both populations. Phenotypic and functional alterations of multiple components of the BM niche have been reported in MPN. Until now, advances in the characterization of the complex ecological system of the normal and leukemic stem cell niche are mainly obtained by experimental studies [70]. In situ imaging of the microenvironment in human samples is often challenging due to the difficulty in preserving integrity of the bone marrow architecture in decalcified trephine BM biopsies and the complexity of immunostaining methods [20]. An important area of future research will be to identify the niche aberrations in MPN in human BM tissue.

New treatment strategies may target not only the malignant clone but also the complex mechanism that governs BMSCs/HSPCs interactions in hematopoietic malignancies.

## References

[1] Seke Etet P. F., Vecchio L., Kamga P. B., Nukenine E. N., Krampera M., Kamdje A. H. N. (2013). Normal hematopoiesis and hematologic malignancies: role of canonical Wnt signaling pathway and stromal microenvironment. *Biochimica et Biophysica Acta: Reviews on Cancer*.

[2] Schepers K., Campbell T., Passegué E. (2015). Normal and leukemic stem cell niches: insights and therapeutic opportunities. *Cell Stem Cell*.

[3] Krause D. S., Scadden D. T., Preffer F. I. (2013). The hematopoietic stem cell niche—home for friend and foe?. *Cytometry Part B: Clinical Cytometry*.

[4] Frenette P. S., Pinho S., Lucas D., Scheiermann C. (2013). Mesenchymal stem cell: keystone of the hematopoietic stem cell niche and a stepping-stone for regenerative medicine. *Annual Review of Immunology*.

[5] Méndez-Ferrer S., Michurina T. V., Ferraro F. (2010). Mesenchymal and haematopoietic stem cells form a unique bone marrow niche. *Nature*.

[6] Ding L., Saunders T. L., Enikolopov G., Morrison S. J. (2012). Endothelial and perivascular cells maintain haematopoietic stem cells. *Nature*.

[7] Ding L., Morrison S. J. (2013). Haematopoietic stem cells and early lymphoid progenitors occupy distinct bone marrow niches. *Nature*.

[8] Méndez-Ferrer S., Scadden D. T., Sánchez-Aguilera A. (2015). Bone marrow stem cells: current and emerging concepts. *Annals of the New York Academy of Sciences*.

[9] Emerson S. G. (2007). Thrombopoietin, HSCs, and the osteoblast niche: holding on loosely, but not letting G0. *Cell Stem Cell*.

[10] Burger J. A., Gribben J. G. (2014). The microenvironment in chronic lymphocytic leukemia (CLL) and other B cell malignancies: insight into disease biology and new targeted therapies. *Seminars in Cancer Biology*.

[11] Keating A. (2012). Mesenchymal stromal cells: new directions. *Cell Stem Cell*.

[12] Bianco P. (2014). ‘Mesenchymal’ stem cells. *Annual Review of Cell and Developmental Biology*.

[13] Li H., Ghazanfari R., Zacharaki D. (2014). Low/negative expression of PDGFR- identifies the candidate primary mesenchymal stromal cells in adult human bone marrow. *Stem Cell Reports*.

[14] Greenbaum A., Hsu Y.-M. S., Day R. B. (2013). CXCL12 in early mesenchymal progenitors is required for haematopoietic stem-cell maintenance. *Nature*.

[15] Omatsu Y., Seike M., Sugiyama T., Kume T., Nagasawa T. (2014). Foxc1 is a critical regulator of haematopoietic stem/progenitor cell niche formation. *Nature*.

[16] Sugiyama T., Kohara H., Noda M., Nagasawa T. (2006). Maintenance of the hematopoietic stem cell pool by CXCL12-CXCR4 chemokine signaling in bone marrow stromal cell niches. *Immunity*.

[17] Bruns I., Lucas D., Pinho S. (2014). Megakaryocytes regulate hematopoietic stem cell quiescence through CXCL4 secretion. *Nature Medicine*.

[18] Nakamura-Ishizu A., Takubo K., Fujioka M., Suda T. (2014). Megakaryocytes are essential for HSC quiescence through the production of thrombopoietin. *Biochemical and Biophysical Research Communications*.

[19] Calvi L. M., Link D. C. (2015). The hematopoietic stem cell niche in homeostasis and disease. *Blood*.

[20] Morrison S. J., Scadden D. T. (2014). The bone marrow niche for haematopoietic stem cells. *Nature*.

[21] Krause D. S., Scadden D. T. (2015). A hostel for the hostile: the bone marrow niche in hematologic neoplasms. *Haematologica*.

[22] Gonzalez-Nieto D., Li L., Kohler A. (2012). Connexin-43 in the osteogenic BM niche regulates its cellular composition and the bidirectional traffic of hematopoietic stem cells and progenitors. *Blood*.

[23] Calvi L. M., Adams G. B., Weibrecht K. W. (2003). Osteoblastic cells regulate the haematopoietic stem cell niche. *Nature*.

[24] Nakamura-Ishizu A., Suda T. (2013). Hematopoietic stem cell niche: an interplay among a repertoire of multiple functional niches. *Biochimica et Biophysica Acta—General Subjects*.

[25] Kunisaki Y., Frenette P. S. (2014). Influences of vascular niches on hematopoietic stem cell fate. *International Journal of Hematology*.

[26] Pennetier D., Oyallon J., Morin-Poulard I., Dejean S., Vincent A., Crozatier M. (2012). Size control of the *Drosophila* hematopoietic niche by bone morphogenetic protein signaling reveals parallels with mammals. *Proceedings of the National Academy of Sciences of the United States of America*.

[27] Luis T. C., Naber B. A. E., Fibbe W. E., van Dongen J. J. M., Staal F. J. T. (2010). Wnt3a nonredundantly controls hematopoietic stem cell function and its deficiency results in complete absence of canonical Wnt signaling. *Blood*.

[28] Bigas A., Espinosa L. (2012). Hematopoietic stem cells: to be or notch to be. *Blood*.

[29] Yamazaki S., Ema H., Karlsson G. (2011). Nonmyelinating schwann cells maintain hematopoietic stem cell hibernation in the bone marrow niche. *Cell*.

[30] García-García A., de Castillejo C. L. F., Méndez-Ferrer S. (2015). BMSCs and hematopoiesis. *Immunology Letters*.

[31] Isern J., García-García A., Martín A. M. (2014). The neural crest is a source of mesenchymal stem cells with specialized hematopoietic stem cell niche function. *eLife*.

[32] Dar A., Schajnovitz A., Lapid K. (2011). Rapid mobilization of hematopoietic progenitors by AMD3100 and catecholamines is mediated by CXCR4-dependent SDF-1 release from bone marrow stromal cells. *Leukemia*.

[33] Arranz L., Sánchez-Aguilera A., Martín-Pérez D. (2014). Neuropathy of haematopoietic stem cell niche is essential for myeloproliferative neoplasms. *Nature*.

[34] Winkler I. G., Sims N. A., Pettit A. R. (2010). Bone marrow macrophages maintain hematopoietic stem cell (HSC) niches and their depletion mobilizes HSCs. *Blood*.

[35] Christopher M. J., Rao M., Liu F., Woloszynek J. R., Link D. C. (2011). Expression of the G-CSF receptor in monocytic cells is sufficient to mediate hematopoietic progenitor mobilization by G-CSF in mice. *Journal of Experimental Medicine*.

[36] Rao M., Supakorndej T., Schmidt A. P., Link D. C. (2015). Osteoclasts are dispensable for hematopoietic progenitor mobilization by granulocyte colony-stimulating factor in mice. *Experimental Hematology*.

[37] Tormin A., Li O., Brune J. C. (2011). CD146 expression on primary nonhematopoietic bone marrow stem cells is correlated with in situ localization. *Blood*.

[38] Lo Celso C., Fleming H. E., Wu J. W. (2009). Live-animal tracking of individual haematopoietic stem/progenitor cells in their niche. *Nature*.

[39] Schepers K., Pietras E. M., Reynaud D. (2013). Myeloproliferative neoplasia remodels the endosteal bone marrow niche into a self-reinforcing leukemic niche. *Cell Stem Cell*.

[40] Omatsu Y., Sugiyama T., Kohara H. (2010). The essential functions of adipo-osteogenic progenitors as the hematopoietic stem and progenitor cell niche. *Immunity*.

[41] Kunisaki Y., Bruns I., Scheiermann C. (2013). Arteriolar niches maintain haematopoietic stem cell quiescence. *Nature*.

[42] Peled A., Petit I., Kollet O. (1999). Dependence of human stem cell engraftment and repopulation of NOD/SCID mice on CXCR4. *Science*.

[43] Coste C., Neirinckx V., Gothot A., Wislet S., Rogister B. (2015). Are neural crest stem cells the missing link between hematopoietic and neurogenic niches?. *Frontiers in Cellular Neuroscience*.

[44] Quick Q., Paul M., Skalli O. (2015). Roles and potential clinical applications of intermediate filament proteins in brain tumors. *Seminars in Pediatric Neurology*.

[45] Krause D. S., Lazarides K., Lewis J. B., Von Andrian U. H., Van Etten R. A. (2014). Selectins and their ligands are required for homing and engraftment of BCR-ABL1^+^ leukemic stem cells in the bone marrow niche. *Blood*.

[46] Lee G.-Y., Kim J.-A., Oh I.-H. (2015). Stem cell niche as a prognostic factor in leukemia. *BMB Reports*.

[47] Tamplin O. J., Durand E. M., Carr L. A. (2015). Hematopoietic stem cell arrival triggers dynamic remodeling of the perivascular niche. *Cell*.

[48] Tabe Y., Konopleva M. (2014). Advances in understanding the leukaemia microenvironment. *British Journal of Haematology*.

[49] Schneider R. K., Ziegler S., Leisten I. (2014). Activated fibronectin-secretory phenotype of mesenchymal stromal cells in pre-fibrotic myeloproliferative neoplasms. *Journal of Hematology & Oncology*.

[50] Kleppe M., Kwak M., Koppikar P. (2015). JAK-STAT pathway activation in malignant and nonmalignant cells contributes to MPN pathogenesis and therapeutic response. *Cancer Discovery*.

[51] Krause D. S., Spitzer T. R., Stowell C. P. (2010). The concentration of CD44 is increased in hematopoietic stem cell grafts of patients with acute myeloid leukemia, plasma cell myeloma, and non-hodgkin lymphoma. *Archives of Pathology and Laboratory Medicine*.

[52] Tabe Y., Konopleva M. (2015). Role of microenvironment in resistance to therapy in AML. *Current Hematologic Malignancy Reports*.

[53] Jin L., Hope K. J., Zhai Q., Smadja-Joffe F., Dick J. E. (2006). Targeting of CD44 eradicates human acute myeloid leukemic stem cells. *Nature Medicine*.

[54] Kim J. A., Shim J. S., Lee G. Y. (2015). Microenvironmental remodeling as a parameter and prognostic factor of heterogeneous leukemogenesis in acute myelogenous leukemia. *Cancer Research*.

[55] Raaijmakers M. H. G. P. (2011). Niche contributions to oncogenesis: emerging concepts and implications for the hematopoietic system. *Haematologica*.

[56] Krause D. S., Fulzele K., Catic A. (2013). Differential regulation of myeloid leukemias by the bone marrow microenvironment. *Nature Medicine*.

[57] Vardiman J. W., Thiele J., Arber D. A. (2009). The 2008 revision of the World Health Organization (WHO) classification of myeloid neoplasms and acute leukemia: rationale and important changes. *Blood*.

[58] Mills K. I., McMullin M. F. (2014). Mutational spectrum defines primary and secondary myelofibrosis. *Haematologica*.

[59] Skoda R. C., Duek A., Grisouard J. (2015). Pathogenesis of myeloproliferative neoplasms. *Experimental Hematology*.

[60] Thiele J., Kvasnicka H. M., Facchetti F., Franco V., Van Der Walt J., Orazi A. (2005). European consensus on grading bone marrow fibrosis and assessment of cellularity. *Haematologica*.

[61] Kröger N., Zabelina T., Alchalby H. (2014). Dynamic of bone marrow fibrosis regression predicts survival after allogeneic stem cell transplantation for myelofibrosis. *Biology of Blood and Marrow Transplantation*.

[62] Schmitt-Gräff A., Skalli O., Gabbiani G. (1989). *α*-smooth muscle actin is expressed in a subset of bone marrow stromal cells in normal and pathological conditions. *Virchows Archiv B Cell Pathology Including Molecular Pathology*.

[63] Shen Y., Nilsson S. K. (2012). Bone, microenvironment and hematopoiesis. *Current Opinion in Hematology*.

[64] Kreipe H., Büsche G., Bock O., Hussein K. (2012). Myelofibrosis: molecular and cell biological aspects. *Fibrogenesis & Tissue Repair*.

[65] Hinz B., Phan S. H., Thannickal V. J. (2012). Recent developments in myofibroblast biology: paradigms for connective tissue remodeling. *American Journal of Pathology*.

[66] Talele N. P., Fradette J., Davies J. E., Kapus A., Hinz B. (2015). Expression of *α*-smooth muscle actin determines the fate of mesenchymal stromal cells. *Stem Cell Reports*.

[67] Xu L., Song C., Ni M., Meng F., Xie H., Li G. (2012). Cellular retinol-binding protein 1 (CRBP-1) regulates osteogenenesis and adipogenesis of mesenchymal stem cells through inhibiting RXR*α*-induced *β*-catenin degradation. *International Journal of Biochemistry and Cell Biology*.

[68] Manshouri T., Estrov Z., Quintás-Cardama A. (2011). Bone marrow stroma-secreted cytokines protect JAK2^V617F^-mutated cells from the effects of a JAK2 inhibitor. *Cancer Research*.

[69] Kvasnicka H. M., Thiele J., Bueso-Ramos C. E. Ruxolitinib-induced modulation of bone marrow microenvironment in patients with myelofibrosis is associated with inflammatory cytokine levels.

[70] Nakamura-Ishizu A., Suda T. (2013). Hematopoietic stem cell niche: an interplay among a repertoire of multiple functional niches. *Biochimica et Biophysica Acta: General Subjects*.

